# Leucocyte esterase dip-stick test as a point-of-care diagnostic for urogenital chlamydia in male patients: A multi-center evaluation in two STI outpatient clinics in Paramaribo and Amsterdam

**DOI:** 10.1186/s12879-016-1946-8

**Published:** 2016-11-03

**Authors:** Menne Bartelsman, Henry J. C. de Vries, Maarten F. Schim van der Loeff, Leslie O. A. Sabajo, Jannie J. van der Helm

**Affiliations:** 1 Department of Infectious Diseases, STI Outpatient Clinic, Public Health Service of Amsterdam (GGD Amsterdam), Weesperplein 1, 1018 XA Amsterdam, The Netherlands; 2 Center for Infection and Immunology Amsterdam (CINIMA), Academic Medical Center (AMC), University of Amsterdam, Amsterdam, The Netherlands; 3 Department of Dermatology, Academic Medical Center (AMC), University of Amsterdam, Amsterdam, The Netherlands; 4 Department of Research, Public Health Service of Amsterdam (GGD Amsterdam), Nieuwe Achtergracht 100, 1018 WT Amsterdam, The Netherlands; 5 Dermatological Service, Ministry of Health Suriname, Tourtonnelaan 5, Paramaribo, Suriname

**Keywords:** Sensitivity, Specificity, Chlamydia, Point-of-care test, Leucocyte esterase test, *Chlamydia trachomatis*

## Abstract

**Background:**

Point-of-care (POC) tests are an important strategy to address the epidemic of sexually transmitted infections (STIs). The leucocyte esterase test (LET) can be used as a POC test for chlamydia. The aim of this study was to determine the diagnostic accuracy of the LET to detect urogenital chlamydia among men at STI clinics in Paramaribo, Suriname and Amsterdam, the Netherlands.

**Methods:**

Recruitment of patients took place in 2008–2010 in Suriname and in 2009–2010 in the Netherlands. Urine of patients was examined with the LET. The reference test was a nucleic acid amplification test (NAAT).

**Results:**

We included 412 patients in Suriname and 645 in the Netherlands. Prevalence of chlamydia in Suriname and the Netherlands was respectively 22.8 and 13.6 %. The sensitivity of the LET was 92.6 % (95 % CI = 85.3–97.0) and 77.3 % (95 % CI = 67.1–85.5) respectively, the specificity was 38.1 % (95 % CI = 32.7–43.6 %) and 58.1 % (95 % CI = 53.9–62.3) respectively. The positive predictive value was 30.6 % (95 % CI = 27.3–36.4) and 22.6 % (95 % CI = 18.0–27.7) respectively and the negative predictive value was 94.5 % (95 % CI = 89.1–97.8) and 94.2 % (95 % CI = 91.1–96.4) respectively. The kappa was respectively 0.179 and 0.176.

**Conclusions:**

To diagnose urogenital chlamydia in men the LET performs poorly. It has a high negative but low positive predictive value. If the LET result is negative, chlamydia is accurately excluded, yet a positive result has a low predictive value. Whether the advantages of direct management based on LET outweigh the disadvantages of overtreatment is a subject for further studies.

## Background

*Chlamydia trachomatis* (Ct) is the most common cause of nongonoccal urethritis in men [1]. In resource-limited settings management of male urethritis is generally based on a syndromic approach: when a patient has typical symptoms like urethral discharge, penile itching or dysuria, immediate treatment with antibiotics is given. Yet, with a syndromic approach, a significant proportion of men infected with Ct are missed, as the majority of men does not have symptoms [2]. The proportion of cases that are asymptomatic varies by population and ranges from 40 to 96 % [3–5]. Moreover, a syndromic approach also can lead to overtreatment, antibiotic overconsumption, induction of side effects and antimicrobial resistance of infections like *Neisseria gonorrhoeae* (Ng). Also, effective contact tracing and treatment is challenging based on a presumptive diagnosis only.

Point of care (POC) management can partly overcome the disadvantages of syndromic management. POC testing is defined as medical testing at or near the site of patient care [6]. Ideally a POC test should meet the ASSURED criteria of the World Health Organization; Affordable, Sensitive, Specific, User-friendly, Rapid and Robust, Equipment-free, Deliverable to those who need them [7]. Recently, several companies have developed commercial POC tests based on bacterial antigens that provide rapid results for the detection of Ct, but the sensitivity of these tests is low (17–65 %) and this precludes more widespread use in clinical settings [6, 8–10]. The Cepheid GeneXpert Ct/Ng assay is a rapid (<2 h to results) nucleic acid amplification test (NAAT) assay that can be performed in on-site laboratories, but the high costs are an obstacle for implementation in low-and middle-income countries. Moreover it is questionable if patients are willing to wait 2 h for their test result [11]. As long as no other promising POC test is available, sexually transmitted infections (STI) clinics are dependent on convential techniques like light microscopy and dipstick urinalysis with the leucocyte esterase test (LET).

Light microscopy is used in the POC management of various STIs [1]. However, light microscopy requires a laboratory infrastructure that is often lacking in resource-limited settings. In these settings the LET might be an alternative for light microscopy, because it is cheap, easy to perform and does not require a laboratory [12, 13]. LET is available as dipstick assay and is used to detect a urinary inflammatory reaction through the presence of an esterase enzyme produced by polymorph nuclear leucocytes (PMNL) in the urine, possibly associated with either a urinary tract infection or STI in males [14].

Suriname is a middle-income country in South America with most of the population concentrated in the coastal region, and the remainder in sparsely populated, predominantly remote areas. For these settings, an equipment-free POC test could be of great benefit. The Netherlands is a high-income country in Europe, but also there an equipment-free POC test may be useful in certain primary health care settings like general practitioners clinics.

Previous studies that investigated the performance of the LET to detect urogenital Ct in males have reported sensitivities and specificities ranging from 46 to 100 % and 60 to 96 % respectively [15–19]. The variety of outcomes can be explained by a difference in settings, patient groups, experimental test thresholds and reference tests. In most of the studies obsolete reference tests like ligase chain reaction (LCR) were used and/or investigated the LET in either symptomatic or asymptomatic patients in one setting [16, 20–28].

The aim of our study was to evaluate the diagnostic performance of the LET as a POC test for urogenital Ct compared to NAAT in male patients irrespective of symptoms attending STI outpatient clinics in two different settings; an outpatient clinic in a middle-income country, Suriname, and in a high-income country, the Netherlands.

## Methods

### Study design, selection of patients

Participants were recruited at:

1. The Dermatological Service in Paramaribo, an integrated outpatient clinic that offers free-of-charge examination and treatment of STIs and infectious skin diseases such as leprosy and leishmaniasis. Recruitment took place between March 2008 and July 2010.
2. The STI Outpatient Clinic of the Public Health Service of Amsterdam, which is a low threshold clinic serving over 40,000 patients annually. Patients were prioritized based on a short questionnaire to estimate the risk of having an STI, as described before [29]. The following categories were regarded as high risk: age <25 years, men who have sex with men, born in an STI and HIV endemic country, receiving money/goods for sex, paying for sex, ≥3 partners in the previous 6 months, reporting a sexual partner with a partner born in an STI and HIV endemic country, being notified by a sex partner or having STI related symptoms. Patients regarded as high-risk were eligible to participate; recruitment took place between November 2009 and May 2010.

In both countries demographic characteristics and data regarding symptoms were obtained using a questionnaire. Patients were regarded as symptomatic when either dysuria, urethral discharge or scrotal pain was reported in the questionnaire.

### Leucocyte esterase test (LET)

The LET (Combur^2^ Test LN, Roche Diagnostics, GmbH, Mannheim, Germany) is a non-specific rapid dipstick assay used to detect the presence of an esterase enzyme produced by PMNL's in urine. The presence of PMNL's in urine indicates an inflammatory response, possibly caused by an infection. In Suriname and the Netherlands patients provided urine samples that were immediately brought to the on-site laboratory by nurses. A laboratory technician performed the LET within two hours of sample collection and placed the dipstick in a urine sample according to the manufacturer’s instructions. The results were read after 2 and 5 min. Using a reference color table, the dipstick differentiated between negative, 1+ (ca. 10–25 PMNL/μl), 2+ (ca. 75 PMNL/μl), 3+ (ca. 500 PMNL/μl). The LET was regarded positive when the dipstick colored ≥ 1+. Patients with a failed or missing LET were excluded from the analyses.

### Reference tests

The Aptima Ct assay (Hologic Gen-Probe, USA) was used as the reference NAAT to detect urogenital Ct both in the Netherlands and Suriname. Reference testing was performed on the same urine sample that was used for the LET. In the Netherlands the samples were tested the same week at the Public Health Laboratory in Amsterdam. In Suriname, the samples were collected according to the manufacturer’s (Hologic) instructions, stored in a fridge (at temperature between 2° and 7 °C) and packed according to international air transport association (IATA) rules for transport by plane to the Public Health Laboratory in Amsterdam for NAAT testing. According to the manufacturer’s instructions the samples had to be analysed within 30 days after collection. In our study 349 of the 412 samples (84.7 %) were analysed within 30 days and 63 samples (15.3 %) were tested between 31 and 48 days (median time: 35 days (IQR 33–36 days)). More details about the data collection were previously described [8, 30].

### Diagnostic performance and statistical analysis

The diagnostic performance of the LET was investigated by calculating the sensitivity, specificity, positive predictive value (PPV), negative predictive value (NPV), the percentage agreement and kappa. A kappa value >0.61 was considered as good test agreement, κ = 0.41–0.60 as fair agreement, κ = 0.21–0.40 as slight agreement, κ = 0.01–0.20 as poor agreement, and κ ≤ 0.00 as no agreement) [31].

As prescribed by the manufacturer the LET was read 2 min after it was placed in urine (from here onwards described as “LET 2 m”). We also read the result of the LET 5 min after it was placed in urine (from now on described as “LET 5 m”) to evaluate the influence of timing on the diagnostic performance.

We compared the diagnostic performance of the LET 2 m between symptomatic and asymptomatic patients and conducted a subgroup analysis for individuals <25 years.

Characteristics of the study populations were compared using the Pearson’s chi-square test. When the expected cell-count was <5 the Fisher’s exact test was used. We considered a *p*-value <0.05 as statistically significant. Analyses were performed with SPSS package version 21 (SPSS Inc., Chicago, Illinois, USA) and STATA software V11.2 (Stata Corp, College Station, Texas, USA).

## Results

### Study populations

#### Suriname

In total 415 patients provided samples for the study, of which three patients had an invalid LET result and were excluded. From the remaining 412 patients 279 (67.7 %) of the patients were symptomatic and 133 (32.3 %) were asymptomatic. The median age was 28 years (interquartile range (IQR) 24.0–37.0). Ct prevalence was 22.8 % (95 % CI 19.0–27.1) (Table 1).

**Table 1:** Characteristics of male patients included at the Dermatological Service Paramaribo, Suriname, 2008–2010 and the STI Outpatient Clinic, Amsterdam, The Netherlands, 2009–2010

	Suriname (*N* = 412)	The Netherlands (*N* = 645)	*P* value
Demographic characteristics	*n* (%)	*n* (%)	
Median age (years) (IQR)	28.0 (24.0–37.0)	34.0 (26.5–43.0)	<0.001
Age (years)			<0.001
< 25	109 (26.5)	110 (17.1)	
25–29	115 (27.9)	123 (19.1)	
30–34	59 (14.3)	108 (16.7)	
≥ 35	129 (31.3)	304 (47.1)	
Symptoms*			<0.001
No	133 (32.3)	379 (59.6)	
Yes	279 (67.7)	257 (40.4)	
HIV status**			<0.001
Negative	392 (95.1)	519 (80.5)	
Positive	2 (0.49)	113 (17.5)	
Unknown	18 (4.4)	13 (2.0)	
Sexual preference*			<0.001
Heterosexual men	402 (98.3)	282 (43.7)	
Men who have sex with men	7 (1.7)	363 (56.3)	
Ct prevalence			
Total population	94 (22.8)	88 (13.6)	<0.001
< 25 years	29 (26.6)	21 (19.1)	0.185
Symptomatic patients*	77 (27.6)	53 (20.6)	0.060
Asymptomatic patients*	17 (12.8)	34 (9.0)	0.207

*Abbreviations*: *STI* sexual transmitted infections; *IQR* interquartile range; *Ct Chlamydia trachomatis*

*Numbers do not add up to column total due to missing data; data on symptoms were missing for 9 patients in the Netherlands and data on sexual preference were missing for 3 patients in Suriname. Percentages were calculated based on those with non-missing data and thus add up to 100 %

**In the Netherlands a positive HIV status was based on a positive HIV test in the past or on the date of inclusion (noted in the patient records); a negative HIV status was based on a negative HIV test result on the date of inclusion. The HIV status was unknown when a HIV test was lacking on the date of inclusion. In Suriname the HIV status was self-reported at the date of inclusion

#### The Netherlands

In total 647 patients provided samples for the study of which two patients had an invalid LET result and were excluded. Data regarding symptoms were missing for nine patients. Among the remaining 636 patients 257 (40.4 %) of the patients were symptomatic and 379 (59.6 %) were asymptomatic. The median age was 34 years (IQR 26.5–43.0). Ct prevalence was 13.6 % (95 % CI 11.2–16.5).

#### Ct prevalence and symptomatology in Suriname and the Netherlands

In Suriname significantly more patients reported symptoms compared with the Netherlands; 67.7 % (279/412) versus 40.4 % (257/636) (*p* < 0.001). Also a significantly higher prevalence of Ct was found in Suriname than in the Netherlands; 22.8 % (94/412) versus 13.6 % (88/645) (*p* < 0.001).

### Diagnostic performance

#### Suriname

With LET 2 m 284/412 had a positive and 128/412 a negative result. The LET 2 m showed a sensitivity of 92.6 % (95 % CI 85.3–97.0), a specificity of 38.1 % (95 % CI 32.7–43.6 %), a PPV of 30.6 % (95 % CI 27.3–36.4), an NPV of 94.5 % (95 % CI 89.1–97.8) and a kappa of 0.179 (Table 2). The LET 5 m had a comparable sensitivity of 96.8 % (*p* = 0.1936) but a significantly lower specificity of 16.7 % (*p* < 0.001). The LET 2 m had a comparable sensitivity among symptomatic and asymptomatic patients, 92.2 % versus 94.1 % (*p* = 0.786), but the specificity was significantly lower among the symptomatic patients compared with the asymptomatic patients; 30.2 % versus 51.7 % (*p* < 0.001).

**Table 2:** Diagnostic performance* of the Leucocyte Esterase Test (LET) after a reading time of 2 min (“LET 2 m”) and 5 min (“LET 5 m”) in the detection of urogenital *Chlamydia Trachomatis* (Ct) among male patients, Dermatological Service in Paramaribo, Suriname, 2008–2010

	N	LET + NAAT+	LET-NAAT+	LET + NAAT-	LET- NAAT-	Sens. (95 % CI)	Spec. (95 % CI)	PPV (95 % CI)	NPV (95 % CI)	Agr.	Kappa
LET 2 m											
ALL	412	87	7	197	121	92.6 % (85.3–97.0)	38.1 % (32.7–43.6)	30.6 % (27.3–36.4)	94.5 % (89.1–97.8)	50.5 %	0.179
Sympto-matic patients	279	71	6	141	61	92.2 % (83.8–97.1)	30.2 % (24.0–37.0)	33.5 % (27.2–40.3)	91.0 % (81.5–96.6)	47.3 %	0.145
Asympto-matic patients	133	16	1	56	60	94.1 % (71.3–99.9)	51.7 % (42.3–61.1)	22.2 % (13.8–32.9)	98.4 % (91.2–100)	57.1 %	0.193
<25 years	109	29	0	47	33	100 % (88.1–100)	41.3 % (30.4–52.8)	38.2 % (27.2–50.0)	100 % (89.4–100)	56.9 %	0.272
LET 5 m											
ALL	412	91	3	265	53	96.8 % (91.0–99.3)	16.7 % (12.7–21.2)	25.6 % (21.1–30.4)	94.6 % (85.1–98.9)	35.0	0.068

*Sensitivity (Sens.), Specificity (Spec.), Positive Predictive Value (PPV), Negative Predictive Value (NPV), Agreement (Agr.) and Kappa

Among patients <25 years of age the sensitivity was 100 % (95 % CI 90.2–100) for the LET 2 m, although this was not significantly different compared with the sensitivity of the those ≥ 25 years; 89.2 % (95 % CI 79.1–95.6) (*p* = 0.066). Also the specificity was not significantly different; 41.3 % (95 % CI 30.9–52.3) versus 37.0 % (95 % CI 30.8–43.4) (*p* = 0.496).

#### The Netherlands

With LET 2 m 301/645 had a positive result and 344/645 had a negative result. The LET 2 m had a sensitivity of 77.3 % (95 % CI 67.1–85.5), a specificity of 58.1 % (95 % CI 53.9–62.3), a PPV of 22.6 % (95 % CI 18.0–27.7), an NPV of 94.2 % (95 % CI 91.1–96.4) and a kappa of 0.176 (Table 3). The sensitivity of the LET 5 m was 90.9 %; significantly higher than the sensitivity of the LET 2 m (*p* = 0.013). The specificity of the LET 5 m was 40.8 %; significantly lower than the specificity of the LET 2 m (*p* < 0.001).

**Table 3:** Diagnostic performance* of the Leucocyte Esterase Test (LET) after a reading time of 2 min (“LET 2 m”) and 5 min (“LET 5 m”) in the detection of urogenital *Chlamydia Trachomatis* (Ct) among male patients, STI outpatient clinic, Amsterdam, The Netherlands, 2009–2010

	N	LET+ NAAT+	LET- NAAT+	LET+ NAAT-	LET- NAAT-	Sens. (95 % CI)	Spec. (95 % CI)	PPV (95 % CI)	NPV (95 % CI)	Agr.	Kappa
LET 2 m											
ALL	645	68	20	233	324	77.3 % (67.1–85.5)	58.1 % (53.9–62.3)	22.6 % (18.0–27.7)	94.2 % (91.1–96.4)	60.8 %	0.176
Sympto-matic patients	257	44	9	100	104	83.0 % (70.2–91.9)	51.0 % (43.9–58.0)	30.6 % (23.2–38.8)	92.0 % (85.4–96.3)	57.6 %	0.208
Asympto-matic patients	379	23	11	129	216	67.6 % (49.5–82.6)	62.6 % (57.2–67.7)	15.1 % (9.8–21.8)	95.1 % (91.4–97.5)	63.1 %	0.026
<25 years	110	18	3	41	48	85.7 % (63.7–97.0)	53.9 % (43.0–64.6)	30.5 % (19.2–43.9)	94.1 % (84.8–98.8)	60.0 %	0.068
LET 5 m											
ALL	645	80	8	330	227	90.9 % (82.9–96.0)	40.8 % (36.7–45.0)	19.6 % (15.9–23.8)	96.6 % (93.4–98.5)	47.6 %	0.125

*Sensitivity (Sens.), Specificity (Spec.), Positive Predictive Value (PPV), Negative Predictive Value (NPV), Agreement (Agr.) and Kappa

The sensitivity of the LET 2 m was not significantly different between symptomatic and asymptomatic patients (*p* = 0.096), but the specificity was significantly lower among symptomatic patients (51.0 %) than among asymptomatic patients (62.6 %) (*p* = 0.008). In the younger age group (<25 years) a high prevalence of Ct was found (19.1 %) but the sensitivity and specificity of the LET 2 m in this group were not significantly different compared with the age group ≥ 25 years (*p* = 0.290 and *p* = 0.377).

Compared with Suriname the sensitivity of the LET 2 m was significantly lower; 77.3 % versus 92.6 % (*p* = 0.004).

## Discussion

To our knowledge this is the first multi-center study that evaluated the LET to detect urogenital Ct among men in clinics in both a middle-income country and in a high-income country with high Ct prevalences (22.8 % and 13.6 %, respectively).

Both in Suriname and the Netherlands the LET 2 m had a reasonably high sensitivity (respectively 92.6 and 77.3 %) but a poor specificity (respectively 38.1 and 58.1 %) and a poor agreement (respectively kappa 0.179 and 0.176). The significantly higher sensitivity of the LET 2 m we found in Suriname compared with the Netherlands can be caused by a difference in study populations. In the Netherlands significantly more patients were asymptomatic (59.6 %) compared with Suriname (32.3 %). Wiggins et al. suggested that asymptomatic patients have infections with a lower infection-load and therefore fewer leucocytes which might cause false negative test results [32]. The same group showed that the number of leucocytes (predominantly PMNL's) was strongly correlated with urethritis and proved that men with asymptomatic urethritis have fewer PMNL's than symptomatic men [33]. A Swedish study conducted among 480 male patients of an STI clinic (also with Ct as outcome and ≥1+ as cutoff point) supports this hypothesis: the LET had a significantly higher sensitivity (69.6 % versus 25.9 %) but a lower specificity (76.3 % versus 85.8 %) among symptomatic patients compared with asymptomatic patients [17].

Moreover, in both countries the LET 2 m showed a significantly higher specificity in asymptomatic patients compared with symptomatic patients. A possible explanation can be that symptomatic patients had relatively more often infections like Neisseria gonorrhoeae (Ng), Mycoplasma genitalum (Mg) or Trichomonas Vaginalis (Tv) compared to asymptomatic patients which could have caused a relative higher proportion of ‘false positive’ results [34–36]. Also in a previous study we showed that light microscopic examination of Gram stained urethral smears is less specific to detect urogenital Ct infections when done in samples of symptomatic males only [37].

In the Netherlands the sensitivity of the LET increased and in both countries the specificity decreased when the incubation time was prolonged from 2 to 5 min. A study of Morré et al. found also a higher sensitivity when the reading time was prolonged from 2 to 5 min [38].

The strength of our study is that we explored the diagnostic accuracy of the LET among both symptomatic and asymptomatic male patients in a high- and in a middle-income country with a different Ct prevalence. A shortcoming is that we only focused on Ct and not on other causative micro-organisms of urethritis like Ng, Mg or Tv. Evaluation of other micro-organisms could have been helped to interpret the difference of false positive results between symptomatic and asymptomatic patients. Another shortcoming is that not all NAAT samples from Suriname were analyzed within 30 days after collection (according to manufacturer’s instructions) at the Public Health Laboratory in Amsterdam; 63 samples (15.3 %) were analyzed between 31 and 48 days, although it is unlikely that this small delay has influenced the performance of the NAAT [39]. Recently, several companies have developed commercial POC tests that provide rapid results for the detection of Ct, however, the sensitivity of these tests is low (25–65 %) and precludes more widespread use in clinical settings [8–10, 40, 41]. An exception of a POC test with a high sensitivity is the GeneXpert Ct/Ng (Cepheid), a cartridge-based automated test that can identify Ct and Ng infections by NAAT within 2 h, but the high costs hinder the implementation in low-and middle-income countries [42]. Moreover, not all patients may be willing to wait for the results for 2 h. Earlier we showed that in the Surinamese setting only 26.7 % of the female STI visitors would be willing to wait for their POC test results for more than one hour [8].

Syndromic management based on symptoms falls short and leaves many asymptomatic infections untreated [40, 43]. As long as no accurate and affordable POC tests for Ct are available, the LET could be a cheap and an easy to perform alternative to exclude urogenital Ct infections among men in settings where any Ct diagnostics are lacking. The sensitivity we found for the LET in Suriname (91.6 %) was reasonably high. It is estimated that a POC test of even moderate sensitivity (63 %) combined with immediate treatment on-site may lead to the treatment of more infected individuals than an ultra-sensitive and specific NAAT alone when patient return is low [44].

Settings lacking any STI laboratory diagnostics now mainly rely on syndromic management for STI treatment. Further studies into the diagnostic performance of the LET test as opposed to routine syndromic management are needed; the effect on overtreatment due to low specificity would be an important end point.

Our previous study on the cost-effectiveness of microscopic examination of Gram stained urethral smears compared to NAAT at the STI outpatient clinic in Amsterdam showed a sensitivity of 83.8 % and specificity of 74.1 %, comparable with the sensitivity and specificity of the LET we found in the current study [37]. However, if available, microscopy is preferable above the LET because it can also detect Ng infections by finding Gram negative diplococci in a Gram stained smear.

## Conclusion

In conclusion, we showed that the LET has a reasonably high sensitivity but a low specificity to diagnose urogenital Ct in male STI clinic visitors. Future studies that also include the detection of other main causative infections of urethritis are needed to compare the cost-effectiveness of the LET in comparison with syndromic management.

## Abbreviations

ASSURED: Affordable, Sensitive, Specific, User-friendly, Rapid and Robust Equipment-free, Deliverable
Ct: Chlamydia trachomatis
IATA: International air transport association
IQR: Interquartile range
LCR: Ligase chain reaction
LET: Leucocyte esterase test
Mg: Mycoplasma genitalum
NAAT: Nucleic acid amplification test
Ng: Neisseria gonorrhoeae
NPV: Negative predictive value
PMNL: Polymorph nuclear leucocytes
POC: Point of care
PPV: Positive predictive value
STI: Sexually transmitted infections
Tv: Trichomonas Vaginalis
